# Recent Advances in Research from Nanoparticle to Nano-Assembly: A Review

**DOI:** 10.3390/nano14171387

**Published:** 2024-08-26

**Authors:** Shamili Bandaru, Deepshika Arora, Kalathur Mohan Ganesh, Saurabh Umrao, Sabu Thomas, Seemesh Bhaskar, Sabyasachi Chakrabortty

**Affiliations:** 1 Department of Chemistry, SRM University AP─Andhra Pradesh, Mangalagiri 522240, Andhra Pradesh, India; shamili_bandaru@srmap.edu.in; 2 Engineering Product Development, Singapore University of Technology and Design (SUTD), 8 Somapah Road, Singapore 487372, Singapore; deepshikha_arora@sutd.edu.sg; 3 Star Laboratory, Department of Chemistry, Sri Sathya Sai Institute of Higher Learning, Prasanthi Nilayam, Sri Sathya Sai, Puttaparthi 515134, Andhra Pradesh, India; kmganesh@sssihl.edu.in; 4 Nick Holonyak Jr. Micro and Nanotechnology Laboratory (HMNTL), University of Illinois at Urbana-Champaign, Urbana, IL 61801, USA; usaurabh@illinois.edu; 5 Carl R. Woese Institute for Genomic Biology, University of Illinois at Urbana-Champaign, Urbana, IL 61801, USA; 6 Department of Bioengineering, University of Illinois at Urbana−Champaign, Urbana, IL 61801, USA; 7 International and Inter University Centre for Nanoscience and Nanotechnology, Mahatma Gandhi University, Kottayam 686 560, Kerala, India; 8 Department of Electrical and Computer Engineering, University of Illinois at Urbana-Champaign, Urbana, IL 61801, USA

**Keywords:** nanomaterials, nano-assembly, self-assembly, nanoscience, nanotechnology, stimuli, oriented assembly, chemical and physical stimuli

## Abstract

The careful arrangement of nanomaterials (NMs) holds promise for revolutionizing various fields, from electronics and biosensing to medicine and optics. This review delves into the intricacies of nano-assembly (NA) techniques, focusing on oriented-assembly methodologies and stimuli-dependent approaches. The introduction provides a comprehensive overview of the significance and potential applications of NA, setting the stage for review. The oriented-assembly section elucidates methodologies for the precise alignment and organization of NMs, crucial for achieving desired functionalities. The subsequent section delves into stimuli-dependent techniques, categorizing them into chemical and physical stimuli-based approaches. Chemical stimuli-based self-assembly methods, including solvent, acid–base, biomolecule, metal ion, and gas-induced assembly, are discussed in detail by presenting examples. Additionally, physical stimuli such as light, magnetic fields, electric fields, and temperature are examined for their role in driving self-assembly processes. Looking ahead, the review outlines futuristic scopes and perspectives in NA, highlighting emerging trends and potential breakthroughs. Finally, concluding remarks summarize key findings and underscore the significance of NA in shaping future technologies. This comprehensive review serves as a valuable resource for researchers and practitioners, offering insights into the diverse methodologies and potential applications of NA in interdisciplinary research fields.

## 1. Introduction

Ever since the advent of nanotechnology and the means to achieve nano-engineering (via top-down and bottom-top approaches), there has been a paradigm shift in the focus of chemists, physicists, and material scientists towards understanding the design principles and functionalities of the broad domain of nano-engineering [1,2,3,4,5,6]. In the past several decades, there has been an exponential growth in the number of reports pertaining to the spectacular sculpting of nano-objects with respect to their intrinsic material properties, size, shape, and local environment (including surface chemistries and functionalization) [7,8,9,10,11]. NMs with myriad structural morphologies such as spheres, rods, plates, tripods, core-shells, cages, tetrapods, ellipsoids, crescents, dumbells, stars, triangles, prisms, and a variety of decorated architectures have been synthesized and evaluated for their optoelectronic functionalities using different simulation softwares (DDA, FDTD, and COMSOL Multiphysics, to name a few) [3,12,13,14,15]. Researchers have been successful in demonstrating high monodispersity, effective solubility, and flexibility in the biofunctionalization of a variety of NMs fabricated using metals, low-dimensional substrates (0D, 1D, 2D), dielectrics (semiconductors and oxides), and salts, as well as polymers [2,8,16]. Over the advent of the third millennium, the research focus regarding the art of nano-engineering has developed tremendous potential for revolutionizing the fields of medical diagnostics, electro-optical devices, non-linear optics, functional wearable materials, smart textiles, drug delivery, and biosensing portfolio [17,18,19,20,21]. It has been observed that such a revolutionary undertaking is explicitly elicited and fostered by the careful engineering of the nano-assemblies that render collective functionalities in an ensemble by virtue of inherent properties, thereby making provision for various tasks concurrently or in progression.

It is instructive to discuss the relevance and significance of self-assembly (SA) and related explorations from the standpoint of initial observations that can be made from the nature we live in. While the macroscopic organization of the cosmos demonstrates the great synergy of SA to sustain an intact planetary motion (devoid of random chaotic interactions), scientists have made important observations of numerous self-assembled molecular machines in living cells that perform functionalities to assist the evolution of different lifeforms [10,11,22,23,24]. At the fundamental level of cognitive understanding, living organisms, especially mankind, appreciate the emergence of order (from chaos) in a particular activity. The cells in the human body mimic the functionalities of the so-called smart materials (counter-intuitively) with diverse innate attributes encompassing self-healing, self-replicating, auto-immunity, and multi-component organization. From an extensive literature review, it has been observed that it is practically not feasible to provide a fixed definition of SA as the terminology is widely used by several scientific disciplines, thereby presenting an infinite flexibility in generalization. However, from a perspective of nano-engineering, which is the topic of the current review, we present a broad definition for SA: ‘the regulated (reversibly controllable) and self-governing union of individual components (or building blocks) from a disordered or less ordered state (at one equilibrium) to a higher order or better-ordered state (with an altered equilibrium state), under the influence of external forces [25,26], so as to realize robust functionalities for a desired application’ [9,24]. The primary requirements to accomplish nanoparticle SA can be listed as follows: (i) NMs that are utilized for obtaining the final self-assembled product, (ii) an adequate understanding of the basic chemical and physical properties of the nanoparticles (NPs) under consideration, (iii) a critical examination of the free energy difference between the reactants (NPs) and the products (nano-assemblies), and (iv) a careful analysis of the interparticle interactions (van der Waals, H-bonding, ionic, electrostatic, magnetic, and molecular) among the NPs used or with that of the template (if incorporated). Importantly, the information that is implicitly coded into the nano-constructs with respect to the shape, size, mass, charge, and intrinsic polarizability (magnetic and electric), established the SA pathway, and realized in the final self-assembled functional units [22,27,28].

While the nano-engineered materials display staggering physicochemical properties that are dramatically different from their bulk counterparts, the engineered nano-assemblies present novel optoelectronic characteristics that are distinctly different from their nanomaterial counterparts [29,30,31,32,33,34,35,36]. Fundamentally, there are two types of SA, namely, (i) static and (ii) dynamic SA. The static SA is a process in which the nano-constructs and the end products are at a particular local or global equilibrium, devoid of the dissipation of energy. Some of the examples of static SA that occur naturally are the specific folding occurring in globular proteins, the pairing of the bases in a cell, and the formation of a lipid bilayer. Further, the generation of atomic, ionic, and molecular crystals as well as liquid and colloidal crystals have been categorized as the static SA, presenting applications in the fields of optoelectronics, displays, molecular sieves, and the development of newer band gap materials. On the contrary, dynamic SA is a process where the individual nano-constructs assemble into higher-order structures with the concomitant dissipation of energy [36,37,38]. A simple example of dynamic SA from the macroscopic standpoint is the association of different forces such as gravity, electromagnetism, and entropy that contribute to the organization of materials in nature around us. Another example that is extensively observed in nature is the indispensable role of SA and associated processes that occur at the cellular level, sustaining the lifeforms on the planet [9,10,28].

Furthermore, different types of classifications of NP SA have evolved in the past decade [28,29,39,40,41]. The general processes involved in the SA formation can be sub-categorized into directed, co-assembly, and hierarchical SA [9,42]. While the directed SA is triggered through an external entity that directs the entire process, the co-assembly is a process where the final nano-assembled constructs are obtained on account of the interactions between two or more NMs of interest that co-assemble in the presence of each other due to the specific physical or chemical interactions between them. Further, the methodology in which the formation of the final desired product occurs via several steps where each step forms the subset of the final self-assembled product comprises the hierarchical SA approach. It is important to note that a different combination of these three categories with the broad classification of static and dynamic SA is possible. For instance, dynamic co-assembly and static hierarchy have been reported in earlier works. Furthermore, depending on the utility of a particular template used in the assembly formation, the processes are categorized as (i) template-independent and (ii) template-dependent. The well-known examples of self-assembled monolayers (SAM) are template-dependent approaches where the SA of NPs is driven on top of the surface/at specific interfaces of interest. The final structural output of the template-dependent SA techniques are highly determined by the liquid–liquid, liquid–air, and liquid–solid interactions at the interfaces of interest/templates [9,28,29]. Such interactions are discussed in detail using the Langmuir–Blodgett method, sedimentation- or evaporation-triggered SA, and the adsorption of NPs at desired surfaces.

The widely accepted classification of SA is based on the external stimuli, which are categorized as physical stimuli and chemical stimuli [23]. While the effect of solvents, acid/bases, metal ions, gases, macromolecules, and redox reaction conditions constitute the chemical stimuli-driven SA, the magnetic field, electric field, light, and temperature-driven methods comprise the physical stimuli technologies. The intrinsic magnetic nature (dia, para, ferro, superpara, ferri, antiferro), dipole–dipole interactions, NP size, and polarizability as well as the chemical functionalities (photo-active ligands) determine the formation of the nano-assembled product in such scenarios [23,42]. In some processes, the conventional nanosynthesis routes are hybridized with the SA approaches to obtain functionally active photo-plasmonic materials. For instance, nanosynthesis may be initiated by the input of light radiation and the NPs formed would immediately SA into ordered structures on account of the interactions of ligand molecules that are capping such NPs. In this case, the activation of ligand molecules to SA, the obtained NPs might be triggered by the input light energy. In other words, the NPs would not self-assemble if they were synthesized via different routes and attempted SA in the dark. Hence, through this example, one can understand that although there are different ways in which the SA of NMs can be classified, by and large, these approaches often overlap. In this background, it is important to note that there are myriad methodologies that are adopted by experimentalists to obtain nano-assemblies. In this context, this review presents an overview of available stimuli-dependent and stimuli-independent approaches for accomplishing nano-assemblies for different applications. Further, we present a focused review on a few state-of-the-art examples encompassing stimuli-dependent approaches and discuss the latest advancements in the domain to cater to the broad audience of nanoscience and nanotechnology research. We believe this review would facilitate further research in the direction of SA by providing new insights, and open new doors to investigate analogous methodologies under the umbrella of stimuli-independent and dependent routes.

## 2. Oriented Assembly of Nanomaterials

Nanotechnology involves the study and manipulation of materials and devices on the nanoscale, typically defined as the scale of 1 to 100 nanometers. Using SA processes to create functional nanoscale materials and devices at interfaces is one component of nanoengineering at available interfaces. SA is a process in which molecules or particles spontaneously arrange themselves into an ordered structure or pattern. This can occur due to various physical and chemical interactions between the molecules or particles, such as hydrogen bonding, electrostatic interactions, and van der Waals forces. SA can occur at different length scales, from the molecular level to the macroscale. Using SA processes for nanoengineering at functional interfaces, we can develop available materials and devices between various materials. This can include interfaces between different types of NPs, between NPs and surfaces, or between different types of surfaces. One of the key advantages of using SA in this context is that it allows for the creation of complex and highly ordered structures with precise control over their size, shape, and composition. This can be achieved by carefully designing the molecules or particles involved in the SA process and the conditions under which SA occurs. Examples of functional materials and devices that can be created by SA at interfaces include sensors, photonic devices, and energy storage and conversion devices. These materials and devices can have various applications, including electronics, biomedicine, and environmental monitoring. Ongoing research in this field is focused on further improving our understanding of SA processes, developing new and more complex functional materials and devices, and exploring new applications for these materials and devices in various fields.

**Figure 1:**
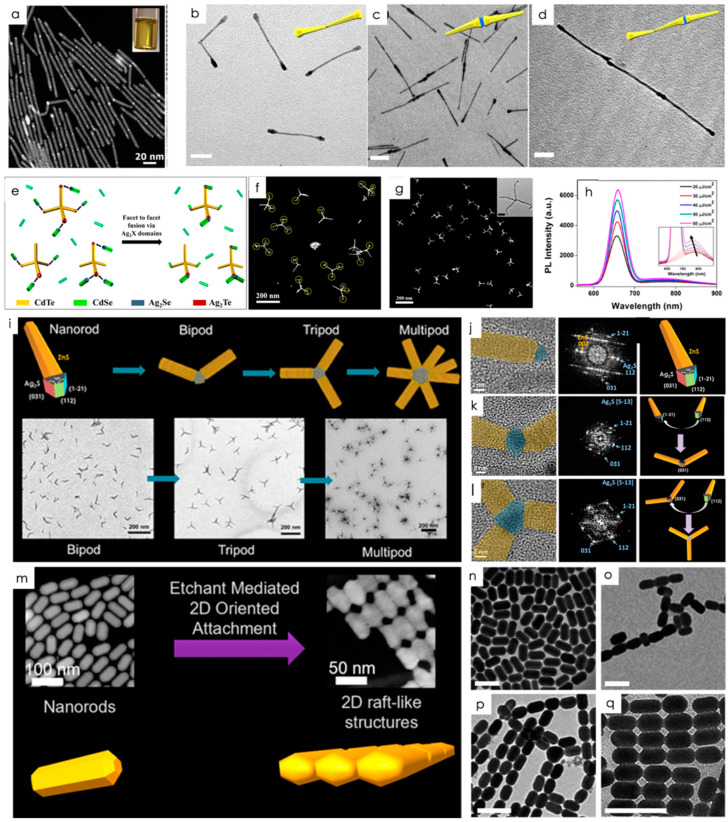
(**a**) Low-resolution HAADF-STEM images of CdSe/CdS nanorods (~50 nm in length and ~4.5 nm diameter) linked by Ag_2_S (~2 nm diameter). Inset shows photograph of well-dispersed, linked nano heterostructures in organic solution without any noticeable aggregation over prolonged times. (**b**) Representative TEM image of typical tail−tail dimers when ~70 nm long asymmetric nanorods were linked by cation exchange with Ag^+^. (**c**) Representative TEM image of head−head dimers linked through cation exchange with Cu^+^. (**d**) TEM image of a hierarchical linked structure comprising three tail−tail dimers joined through their head ends, fabricated by sequential cation exchange with Ag^+^ and Cu^+^. TEM images of hybrid structures formed by using Ag^+^ to link. These images were cited and reproduced from the reference [43]. (**e**) Schematic showing hetero-linking of Ag_2_Se-tipped CdSe NRs (green) to Ag_2_Te-tipped CdTe TPs (yellow) via their Ag chalcogenide ends. Due to competitive cation exchange, not all CdSe NRs undergo exchange with Ag^+^ and remain as pristine NRs. (**f**) HAADF-STEM image of CdSe NR-linked CdTe TPs. The dotted circles denote the linkage regions. (**g**) HAADF-STEM image of CdSe NR-linked CdTe TP nanostructures after Cd^2+^ back-exchange and size-selective precipitation. (Inset) Typical Cd^2+^ back-exchanged CdTe−CdSe (TP−NR) HtNS. The scale bar is 20 nm. (**h**) Pump-fluence-dependent PL spectra of linked nanostructures dispersed in toluene. (Inset) Zoomed-in image of PL spectra, showing the evolution of the type II emission at different pump intensities. These images were cited and reproduced from the reference [46]. (**i**) Schematic illustrating the formation of branched heterostructures with a controlled number of branches as well as orientation. (**j**–**l**) Mechanism of formation of bipods and tripods. These images were cited and reproduced from the reference [47]. (**m**) Schematic illustrating the fabrication of 2D Rafts from NRs. (**n**–**q**) Representative TEM images showing the various stages of 2D raft formation (**n**)NRs, (**o**) chains of NRs, (**p**) Longer chanins of NRs, and (**q**) 2D Rafts [48].

While SA obtained via simple solvent evaporation and gravitational sedimentation are classic examples of stimuli-independent techniques, in this section we discuss oriented attachment, which is considered one of the important categories. In oriented attachment, the NPs are attached to each other, making it an attractive method of assembling NPs. As a result of oriented-assembly (OA), multiple crystalline semiconductor nanocrystals (NCs) are aligned and brought into contact with each other in a solution, resulting in the fusion of them into a single crystal. The nature of OA differs from SA in the way that NCs are in proximity but not necessarily atomically bound in the case of SA. The dispersion of NCs can be facilitated by surface-functionalizing them with ligands. NCs can be conjoined by replacing specific facets with labile ligands or removing them by adding antisolvents. Through this process, larger nanostructures with simple or complex geometries are formed which cannot be formed using homogeneous or heterogeneous nucleation and growth alone. Through OA, it is possible to fabricate SAM NMs with new structures and functions by combining multiple NPs into a single assembly. A geometrically regular assembly of electronically coupled NPs with long- and short-range orders can be manufactured using this method. Although complex, shape-anisotropic NPs pose challenges when it comes to balancing particles, ligands, and solvents, Chan et al. have demonstrated a method involving OA that involves the formation of reactive inorganic intermediates through site-selective nucleation to covalently join elementary NPs, thus eliminating the need to modulate weak interparticle interactions. The method can be used to join particles of numerous shapes, including symmetric nanorods (NRs) and tetrapods (TPs). A variety of structurally complex discrete structures and polymer-like chains are possible via this methodology. Nucleating the intermediate and spontaneously linking it together in stoichiometric fashion is capable of fabricating nanostructures with arbitrarily complex geometries. For instance, uniform dodecylamine (DDA)-capped CdSe/CdS NRs were fabricated and linked via their ends, as illustrated in the representative high-angle annular dark-field scanning transmission electron microscopy (HAADF-STEM) image in Figure 1a [43]. These structures are solution-processable and possess high colloidal stability (see Inset Figure 1a). NRs are activated by cation exchange reactions with Ag^+^, resulting in small regions of Ag_2_S only at the rod tips. Next, an acid–base reaction is carried out using octadecyl phosphonic acid (ODPA), resulting in an insoluble salt with DDA. This process involves removing ligands from Ag_2_S surfaces and linking NRs together into long chains. Domains of Ag_2_S are the only ones involved in linking. Due to the stoichiometric and site-selective nature of the linking process, particle ordering can be controlled. The linkage sites are determined by the positions of the Ag_2_S domains. The final assembly can be controlled by modifying the driving force for their nucleation. Due to the curvature of NR ends, reducing the curvature greatly lowers the barrier to heterogeneous nucleation [44,45]. This is because the critical nucleus size is proportional to the curvature of the NR ends. To illustrate this effect, more complex particles were utilized. Matchstick-shaped NRs, for example, have bulged heads at one end and thin tails with different curvatures at the other. Cation exchange with Ag^+^ results in the nucleation of Ag_2_S only at the tail end of Ag_2_S. The asymmetric NRs, however, produced dimers only when exchanged with Ag^+^, as shown in Figure 1b.

Nucleation can also be affected by the chemical composition of the linkages. Inorganic intermediates were created by performing a partial exchange of Cu^+^ with Cu_2_S domains [44] since Cu-based chalcogenides can also attach through oriented attachment. The high reactivity of Cu^+^ led to the exchange of both ends of the asymmetric NR, forming small Cu_2_S domains with higher and lower curvatures. As Cu^+^ is incorporated into Cu_2_S domains, the driving force for cation exchange gradually decreases, ultimately leading to preferential dissolution at the tail-end region due to size-dependent solubility and resulting in mainly epitaxial head-to-head linking (Figure 1c). For building structural complexity, inorganic intermediates are an excellent synthetic strategy. For example, the Cu^+^-based linkage of tail-to-tail linked dimers is capable of secondary joining, resulting in an orderly arrangement of asymmetric NRs (Figure 1d) [46,49].

It is essential to join NPs in a stoichiometrically controlled manner to form heterostructures without forming links between NPs with the same composition. Conventional methods of attachment pose an inherent challenge. A facile method of joining colloidal semiconductor NRs of different shapes and compositions in a stoichiometrically controlled manner was conducted by linking CdTe TPs to CdSe NRs in solution. The fabrication of complex semiconductor nano heterostructures was simplified by synthesizing CdTe TPs and CdSe NRs separately, then combining them through partial cation exchange. The CdTe TPs and CdSe NRs form small domains of Ag_2_Te and Ag_2_Se after partial cation exchange. After the surface ligands have been selectively removed from the Ag chalcogenide surfaces, a cohesive bond is formed when the unpassivated surfaces meet. Because many of the NRs in Ag_2_Te-tipped CdTe TPs haven’t been exchanged for Ag^+^, they are sterically prevented from joining, enabling heterolinking with Ag_2_Se-tipped CdSe NRs. TPs and NRs are linked spontaneously by the fusion of Ag_2_Te-tipped CdTe TPs and Ag_2_Se-tipped CdSe NRs, ensuring electronic coupling between TP and NR [50]. Due to the energetic favorability of the process, bare silver chalcogenide facets form numerous covalent bonds with the CdSe NRs at the tips of CdTe TPs. Based on the HAADF image (Figure 1g) of the NR-joined TPs, it is evident that several hetero nanostructures are formed via joining CdTe TPs with CdSe NRs, resulting in hetero-linked CdTe−Ag_2_Te/Ag_2_Se−CdSe structures. Unlike pristine CdSe NRs, homo-linked CdSe NR chains are rare, due to the relatively small proportion of CdSe NRs that undergo Ag^+^ exchange. It was also rare to find chains or free TPs that were homo-linked. Heterostructures can thus be produced very effectively through the exemplified technique. As illustrated in Figure 1f, the morphology of the converted CdTe−CdSe TP−NR structure is preserved, as expected from cation-exchange reactions carried out at room temperature. The PL spectrum shifts blue with increasing pump fluence, as shown in Figure 1h. Consequently, this observation relates to a Coulombic interaction between electrons and holes separated by space [51,52,53,54].

These works of controlled OA were advanced by Arora et al.; by linking non-spherical semiconductor nanoparticle building blocks facet-to-facet, they were able to synthesize branched nanostructures, which is inherently different from the traditional process of heterogeneous nucleation and growth. The principle of facet-to-facet linking was illustrated for the first time using monoclinic Ag_2_S-seeded ZnS NRs [47]. Nanoparticle OA creates bipods and tripods by covalently linking the facets of proximal Ag_2_S NPs with ZnS NRs with Ag_2_S tips. As a result of gold deposition experiments, it was concluded that the sides of the Ag_2_S tip have more S than Ag, resulting in angular rather than linear structures. A 120° edge facet orientation was also inferred from high-resolution electron microscopy data (Figure 1j). Finally, they modeled the Ag_2_S atom distribution to determine that side facets of two Ag_2_S-tipped ZnS NRs were indeed rich in S. On the other hand, they found that apex facets were Ag-rich, explaining why they fuse at 120° to form bipods and tripods. The cation-exchange method effectively controlled the orientation of attached NPs, leading to the formation of well-defined nanostructures.

OA of semiconductor NCs to 1D and 2D nanostructures with unique properties can fabricate quantum-confined NMs, which are otherwise difficult to synthesize directly. It is challenging to link 1D NCs, such as NRs, in a faceted-specific manner to produce 2D structures because OA creates linear chains, rod-couple structures, or clustered columns. One of the recent works has reported that 1D Cu_2−x_S NRs undergo etching on exposure to hexylphosphonic acid (HPA) under mild heating [48]. Due to this, the surface ligands at those sites are reduced, and the curvature becomes more pronounced. NRs form chains joined by their tips and are fused via their diametrically opposed sides to form atomically coupled 2D rafts as shown in Figure 1m. Stepwise OA demonstrated here allows solution-processed methods to produce a larger range of nano architectures. Two-dimensional raft-like structures are formed by NRs whose thickness is identical to an individual NR. This process occurs when NRs containing t-dodecanethiol (t-DDT) and oleylamine (Olam) are exposed to toluene containing high concentrations of HPA. Numerous characterization techniques have shown that the tips of the NR etch faster than the sides, and that its crystal structure changes from monoclinic to triclinic when it is subjected to such reaction conditions. Apparently, HPA preferentially extracts Cu^+^. Chains are formed from the tip to the tip (Figure 1p), and raft-like structures (Figure 1q) are formed from the lateral fusion of diametrically opposed sides. OA in 2D is indicated by an epitaxial registry between the attached tip and side facets. The starting NRs were analyzed using a selected area electron diffraction analysis (SAED) in order to clarify why they connect laterally rather than cluster up three-dimensionally. We determined three unique side faces with varying atomic compositions. After HPA removes the Cu^+^, the side facets of the rod are anisotropically etched, resulting in a slightly flattened rod shape with two sides of maximum curvature, suggesting the possibility of lateral fusion into raft-like structures. These works have demonstrated the potential of the cation-exchange method for achieving oriented attachment of NPs and the formation of complex nanostructures with enhanced properties. Further, these works have opened new avenues for developing advanced NMs and provided insights into orientation mechanisms.

## 3. Stimuli Dependent Techniques

### 3.1. Chemical Stimuli-Based Self-Assembly of Nanomaterials

#### 3.1.1. Solvent-Induced Self-Assembly

The colloidal stability of NPs is immensely influenced by the characteristics of their ligand passivation dynamics, which in turn is affected by the nature of the solvent. Some solvents may cause the NPs to be colloidally stable, while others may cause them to aggregate (controlled or uncontrolled) in the solution. In general, the addition of an immiscible solvent (also known as a bad solvent) to a colloidal NP solution can trigger SA of the NPs, as the immiscible solvent causes the surface ligand molecules to switch between stabilized and destabilized states. This process reduces the steric repulsion between ligands, leading to NP aggregation. At large, the controlled aggregation may lead to self-assembled nano-architectures [55,56]. Although solvent-driven assembly is generated through non-covalent interactions, it can be easily disrupted by removing the stimuli (immiscible solvent) or by introducing competing stimuli (miscible solvent). Thus, the solvent-induced SA approach relies on the interplay between immiscible and miscible solvents to achieve the controlled aggregation of NPs.

**Figure 2:**
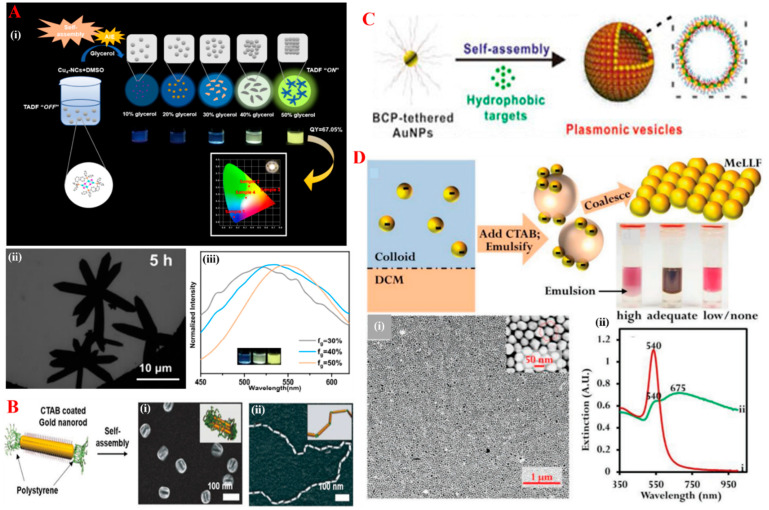
(**A**) Solvent-assisted self-assembly of Cu NCs with improved quantum yield due to their aggregation with the addition of anti-solvent glycerol to the Cu NCs in DMSO solution, (**i**) overall schematic followed in this approach, (**ii**) TEM image of self-assembled Cu NCs, (**iii**) and the change in their fluorescent spectra of NCs before and after self-assembly. These images were cited and reproduced from the reference [57]. (**B**) Organization of Au nanorods having distinct ligands on the long and short ends and their self-assembly of (**i**) side-by-side and (**ii**) end-to-end patterns when exposed to stimuli in the form of anti-solvent. These images were cited and reproduced from the reference [61]. (**C**) Self-assembly of block co-polymer-stabilized Au NPs in the form of vesicular structures via emulsification in chloroform and dodecyl sulphate solution. These images were cited and reproduced from the reference [63]. (**D**) Promoter- or modifier-assisted self-assembly in oil–water interface, (**i**) TEM image of the metal liquid-like layer NPs assembly, (**ii**) and optical spectra for the obtained self-assembly. These images were cited and reproduced from the reference [64].

In a classical example, the use of a solvent-assistant SA route can improve the quantum yield of Cu nanoclusters (NCs). Initially, 1.8 nm-sized triphenylphosphine-capped Cu NCs were synthesized in DMSO (dimethyl sulfoxide) solvent, to which an immiscible solvent (typically glycerol, a bad solvent) was introduced to induce the SA of Cu NCs, as shown in Figure 2A (top (i)). Upon introducing glycerol to the stable phosphine Cu NCs-DMSO solution, Cu NCs started aggregating in the DMSO-Glycerol system. As the glycerol concentration increased in the DMSO-Glycerol system, Cu NCs aggregated and precipitated in highly ordered structures with branched morphologies, due to the stacking of ligands and non-covalent interactions with adjacent Cu NCs in an end-to-end manner (Figure 2A (down left (ii))) [57]. Additionally, a higher amount of glycerol resulted in a change in the final morphology, with an increase in the size of Cu NCs, as well as a strong yellow-green emission, which is depicted in Figure 2A (down right (iii)).

The manipulation of NPs that have a heterogeneous chemical nature and different facet reactivities could lead to another strategy for the solution-SA. Here, non-spherical NPs are mostly involved in assembled nanoarchitectures. In particular, side-by-side and end-to-end assemblies of NRs were obtained by triggering the interactions among distinct stabilizing agents upon the addition of stimuli [58,59]. Generally, they are in the form of anti-solvents [60]. For example, the SA of gold NRs stabilized with CTAB (cetyl trimethyl ammonium bromide) on the long facet and PS (polystyrene) in the short facet manifest two distinct assemblies depending on the solvent used, as shown in Figure 2B. In a water-tetrahydrofuran (THF) system, the presence of THF acted as an anti-solvent for CTAB that reduced the steric repulsions on longer facets of NRs. As a result, it triggered the side-by-side interactions and Individual Nanorods self-assembled to bundle shaped structures, as shown in Figure 2B (left (i)). On the other hand, in the dimethylformamide–water system, where water was treated as an anti-solvent for PS, end-to-end interactions of gold nanorods were observed. As shown in Figure 2B (right (ii)), the SA in the form of a long chain interconnected with the PS–PS short facets was present [61]. Additionally, the assembly of NPs obtained at liquid–liquid interfaces offer an efficient pathway to build up multi-dimensional arrays. In particular, the SA of amphiphilic NPs at a liquid–liquid interface is a well-known approach [62]. In a typical example, amphiphilic Au NPs were synthesized and dispersed in chloroform, where they were stabilized by a block co-polymer (polystyrene-b-poly(ethylene oxide)) ligand. Then, sodium dodecyl sulphate was introduced to allow the emulsification process that resulted in the vesicular SA of plasmonic NPs as shown in Figure 2C. A change in the quality of SA also differed with respect to the length of block co-polymer and the size of Au NPs [63]. In addition, the SA of NPs at oil–water interfaces can be obtained by the addition of promoter or modifier. In a typical example, Chunchun Li and co-workers used CTAB as a promoter and acquired a multi-dimensional SA of metal liquid like thin layer of Au NPs at the oil–water interface [64] as shown in the Figure 2D(top). Changes in the optical properties shown in Figure 2D (down right (ii)) and the TEM image Figure 2D (down left (i)) evidenced the SA of citrate-capped Au NPs at the DCM (dichloromethane) and water interface.

#### 3.1.2. Acid–Base-Induced Self-Assembly

**Figure 3:**
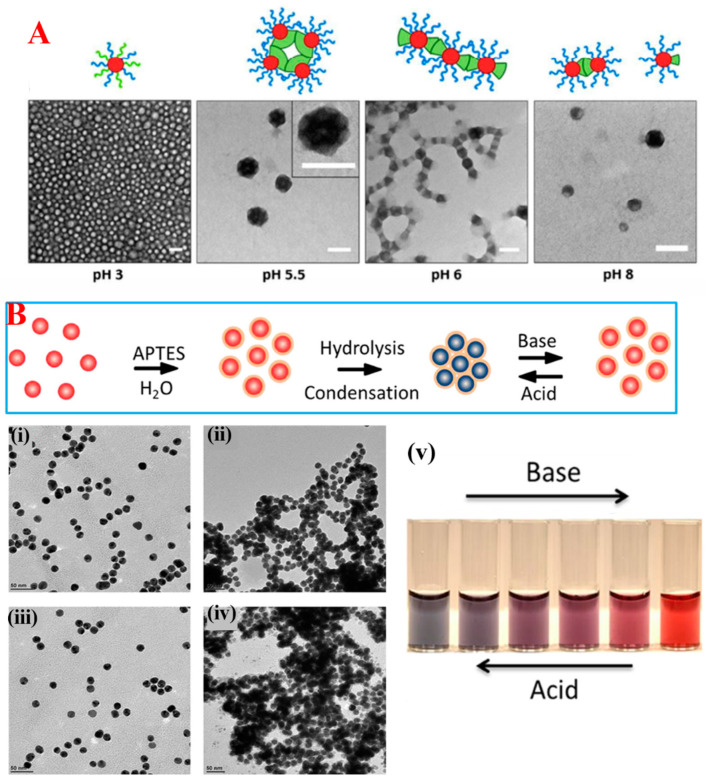
(**A**) pH-responsive polymer-driven self-assembly of terpolymer block particles at different pH levels. These images were cited and reproduced from the reference [65]. (**B**) Assembly and disassembly of citrate-capped Au NPs in the presence of APTES with the supplementation of acid and base to the medium. (**i**–**iv**) TEM images of Au NPs, self-assembled Au NPs in APTES, disassembly of Au NPs when pH changes from 9.8 to 12, and retained assembly when pH decreased from 12 to 9.8 pH. (**v**) Digital image of the visible color change with respect to self-assembly and disassembly. These images were cited and reproduced from the reference [66].

Stabilization of colloidal NPs purely relies on the surfactant molecules where they covalently or coordinately bonded to the NPs surface atoms. The terminal groups of ligands dictate the possible interaction depending on their charges. Electrostatic repulsions between the highly negatively charged surfaces or highly positive surfaces of NPs are responsible for the stability of NPs in an aqueous medium. Thus, modifying the solution pH, the protonation/deprotonation of pH-responsive terminal groups of ligands initiate the aggregation of NPs. Recently, Alberto Piloni and co-workers developed a caterpillar-shaped controlled SA of triblock glycopolymers with the assistance of a pH-responsive poly(4-vinyl pyridine) (P4VP) core [65]. In the first step, a hydrophobic PBuA-P4VP core triblock terpolymer system with hydrophilic glycopolymer block spherical core/shell micelles were formed in water. While changing the pH, P4VP triggered the hierarchical SA of terpolymer via the aggregation of micelles instead of collapsing the P4VP block through ionization and deprotonation processes. TEM images of the respective changes with respect to pH are shown in Figure 3A. Generally, this approach is regulated by supplying the promoter or deactivator in the form of an acid or base with respect to the pH-responsive ligand. In another example [66], Yun Liu and co-workers studied the assembly and disassembly of citrate-capped Au NPs in the presence of 3-aminopropyltriethoxysilane (APTES) with the addition of NaOH and HCl as a promoter and deactivator. In the presence of APTES, Au NPs self-assembled with a visible color change from wine red to purple as shown in the Figure 3B (down right (ii)) digital image. Self-assembled Au NPS undergo disassembly in the presence of supplement base (NaOH), likely due to the deprotonation of amino acids in (3-Aminopropyl)triethoxysilane (APTES) causes visible color change from purple to red. In contrast, acid treatment protonated the amino acids and retained the SA of these Au NPs. The respective TEM images are shown in Figure 3B (down (i–iv).

#### 3.1.3. Bio-Macromolecule-Induced Self-Assembly

**Figure 4:**
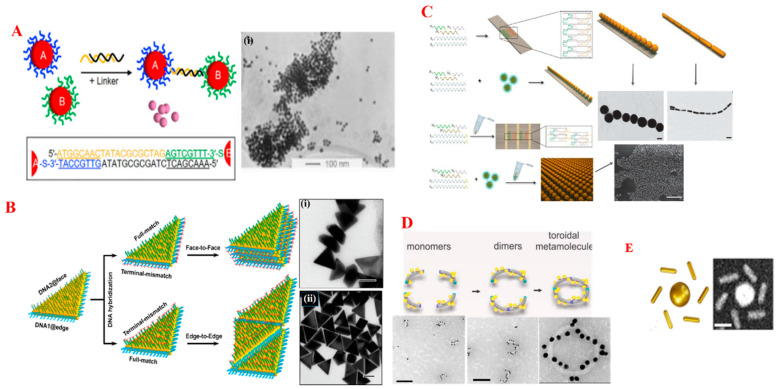
(**A**) An illustrative example of the DNA-driven self-assembly where the surface of NPs functionalized with single-strand DNA and induced self-assembly via complementary linker. This image was cited and reproduced from the reference [68], (**i**) shows the respective TEM image of SA NPs. (**B**) DNA-driven regioselective self-assembly in side-by-side and end-to-end manner induced by matched and mismatched oligonucleotides. (**i**,**ii**) are the respective TEM images. These images were cited and reproduced from the reference [69]. (**C**) DNA origami approach for the synthesis of 2D and 3D Au nanostructures. This image was cited and reproduced from the reference [70]. (**D**,**E**) are the classical examples for the origami mediated self-assembly. These images were cited and reproduced from the reference [71,72].

In geometric terms, a template is a substrate with a pre-defined active surface. Template-directed SA is recognized as an ideal approach for tuning the shape and chirality of NPs in a pre-defined order. Strong interactions between the functionalized NPs and multiple well-defined binding sites of the template direct the assembly of NPs in a pre-specific manner. With this concept, a diversity of classes of templates such as block co-polymers, macromolecules, and single molecules have been used in manipulating the organization of NPs. Due to their spatial distribution of reactive sites and great affinity for specific particle deposition control, the hierarchical structures on soft templates (bio-macromolecules) are advantageous compared to hard templates (carbon tubes). Bio-macromolecule-induced (particularly deoxyribonucleic acid (DNA)-driven) assembly is a promising and attractive approach in the development of novel assembled NMs. Their multi-functional binding sites with a phosphate backbone provide strong interactions with metals for binding [67]. The precise organization of DNA strands offers a pre-eminent technique to generate various complex nanostructures. DNA technology-based SA can be divided in two approaches. In the first case, metal NPs were functionalized with single or several Immobilized DNA strands and allowed to aggregate in a cross-linking manner in the presence of complementary linker [68], schematic of cross-linking manner was shown in Figure 4A (right) and the respective TEM was shown in the Figure 4A (left(i)). This process was deliberately employed to generate various shapes and ordered structures. Apart from this, short DNA strands are artificially modified by changing the bases and the insert/removal of specific sites to obtain the complex SA of NPs. However, immobilized regioselective decoration on DNA strands is one of the advanced processes in DNA-templated SA technology. Long and short facets of variously shaped NPs were intentionally decorated with differently sequenced oligonucleotide DNA strands. Subsequent supplementation of matched and mismatched complementary linkers allowed the NPs to form duplexes and assemble in a side-by-side (as shown in Figure 4B(i)) or end-to-end manner (as shown in Figure 4B(ii)) [69] that was largely dependent on the complementary linker, as shown in Figure 4B (right schematic).

The second approach is the SA via DNA origami where the shape of the template is precisely pre-modified with short and long DNA strands having active surfaces that provide binding sites to NPs. In a typical example, thio-DNA-functionalized Au NPs of five different sizes were self-assembled on the ribbon-like DNA origami as shown in Figure 4C. However, 1D and 2D SA can be controlled by short and folded DNA strands [70]. Figure 4D,E represents the various complex structures synthesized via DNA origami technology.

In addition to DNA biomolecules, bio-polymers play significant role in the SA of NPs. The utilization of bio-polymers such as proteins, peptides, nucleotides, and synthetic biopolymers has become a reliable approach in SA techniques to create well-defined nanostructures [73,74]. Owing to their low toxicity, synergistic functionality, biocompatibility, and multiple levels of structural complexity, bio-polymers are a promising candidate to engineer self-assembled NMs for drug delivery and biosensor applications for the detection of various biomolecules. The inherent ability of biopolymers to fold into complex structures enables the precise organization of NPs through various interactions, including electrostatic forces, hydrogen bonding, hydrophobic interactions, and covalent bonding. These interactions facilitate the creation of stable, functional NP-biopolymer assemblies with tailored properties [75]. For example, hierarchical structures of graphene quantum dots were developed by integrating graphene quantum dots with biopolymers for biosensor applications. pH-responsive structural changes in bio-polymers, availability of binding sites for specific biomolecule conjugation, and enzymatic hydrolysis made the graphene quantum dot-biopolymer-integrated system a versatile biomaterial with multifunctional characteristics [76]. Therefore, it is anticipated that besides DNA functionalization on NPs, the integration of bio-polymers is also a prominent technique that can be applicable in a vast range of applications ranging from biomedicine to electronic device fabrication.

#### 3.1.4. Metal Ion-Induced Self-Assembly

**Figure 5:**
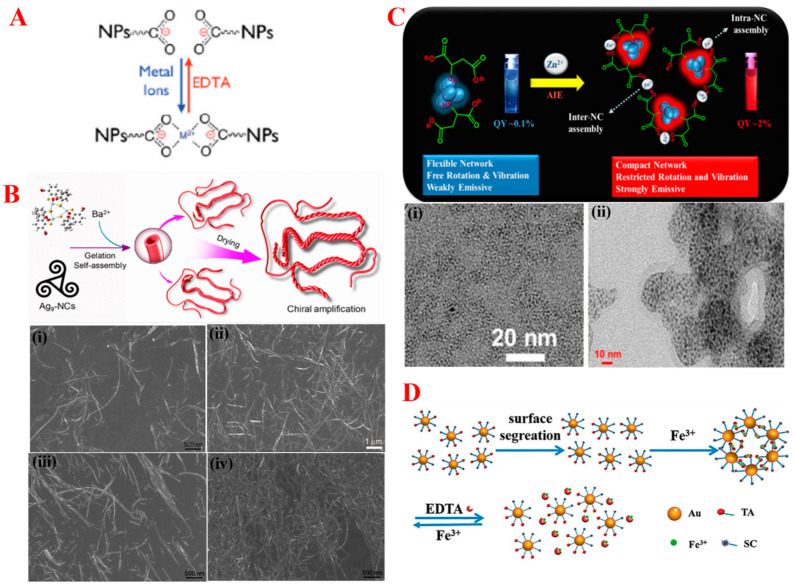
(**A**) Represents the illustration of metal ion coordination with ligand carboxylate group. This image was cited and reproduced from the reference [79]. (**B**) (top) schematic illustration of Ag NCs self-assembly into nanotubes when coordinated with Ba^2+^. (**i**–**iv**) (down) are the TEM images at various concentrations (20 mmol·L^−1^, 40 mmol·L^−1^, 60 mmol·L^−1^, 100 mmol·L^−1^) of Ba^2+^ ion. These images were cited and reproduced from the reference [80]. (**C**) (**i**,**ii**) depict the TEM images of Ag NCs before and after the addition of Zn^2+^. These images were cited and reproduced from the reference [81]. (**D**) represents the schematic of site-selective metal co-ordinate self-assembly process of Au NPs. This image was cited and reproduced from the reference [82].

Transition metal ion-mediated SA is another appealing approach in synthesizing various geometries of nanostructures. Since they express a wide range of optical, electronic, magnetic, radioactive, and catalytic properties, metal ion-induced stimuli becomes one of the ideal approaches for using them in later applications [77]. Generally, metal ions act as a vertex of polyhedrons with the attached ligand of NPs. Carboxylate groups of organic ligands usually serve as active binding sites for the metal ion interaction. In short, during the SA approach, metal ions and the surface ligand of NPs were spontaneously bound together via coordinate bonding as a mean of a Lewis acid/base interaction [78]. As shown in Figure 5A, where metal ions (M^2+^) interact with two or more active carboxylate groups of surface ligands, NPs are closer as a result of SA. A balanced series of interactions of attractive and repulsive forces ease the disassembly of NPs that could be promoted with the addition of a strong chelator such as EDTA [79]. Recently, hydrogels of Ag NCs supramolecular structures were self-assembled with the help of a Ba^2+^ secondary metal ion. The orientation of enantioselective carboxylic groups promotes the assembly of NCs into nanotubes when coordinated with Ba^2+^ as shown in Figure 5B (top). Different concentrations of secondary metal ions were investigated during the formation of Ag nanocluster hydrogels where intertwining increases with the increase in the concentration of Ba(NO_3_)_2_ as shown in Figure 5B (down (i–iv) SEM images) [80]. In another example, the optical properties of Au nanoclusters were improved via the metal ion coordination approach. Paritosh Mahato and co-workers observed the red shift in PL λ_max_ and increase in PLQY from 0.1% to 2% with the addition of Zn^2+^ cations as shown in Figure 5C. This is due to the restriction on rotation and vibration motions of the compact network surface ligands, which resulted in an increased radiative relaxation through a metal-centered triplet state [81]. As shown in Figure 5C (down (i,ii)), TEM images evidence the aggregation of Au nanoclusters with the addition of Zn^2+^ cations. However, NPs self-assembled with the addition of cations unfortunately neutralize charges and simultaneously result in the precipitation or uncontrolled growth of NPs; this result was very common. Therefore, controlling the SA of NPs during an ion-responsive stimuli process would be very important. Recently Jiao Su and co-workers studied the controlled dynamic SA of NPs with the help of mixed ligands as shown in Figure 5D. In a typical example, with the addition of Fe^3+^ cations to 5 nm-sized Au NPs, they underwent SA to 50 nm quasi-spherical superstructures. However, due to the phase separation of the tannic acid and citrate ligand on the surface, Au NPs restricted the additional growth and addition of strong metal chelator EDTA, which resulted in the disassembly of NPs [82]. Furthermore, the assembly of NPs in an ion-induced approach could be highly directional for site-selective strategies. Recently, Binghui Wu and co-workers studied the anisotropic SA of Au/TiO_2_ NPs through a site-selective approach. In this work, they have studied the effect of surface ligand density on the morphology of Au NRs. A nanodumbell-shaped assembly of NPs was acquired due to the large deposition of TiO_2_ at the lower density and higher curved tip of Au NRs [83].

#### 3.1.5. Gas-Induced Self-Assembly

**Figure 6:**
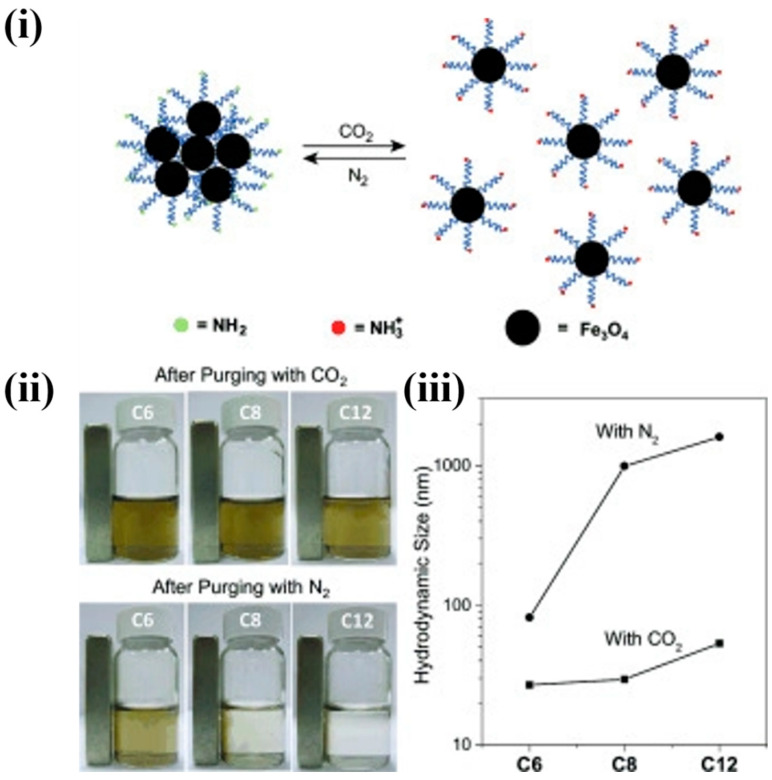
(**i**,**ii**) Represents the schematics and digital image of the iron oxide NPs (when purged with CO_2_ and N_2_). (**iii**) Change in hydrodynamic size of iron oxide NPs caused due to presence of CO_2_ and N_2_. These images were cited and reproduced from the reference [84].

Since gaseous substances could be easily removed from the reaction system through sonication, purging, or heating, vapor-induced assembly is likely to be a unique stimulus in NPs SA. Here, the assembly and disassembly of NPs can be easily tuned by a gassing and degassing process. Amongst all other gases, CO_2_ was an exceptionally desirable candidate in gas stimuli-induced SA because of its biocompatibility and reactivity with the surface ligands. Amino group-functionalized NPs show more responsiveness towards this kind of SA because of its indirect deprotonation/protonation with the addition of CO_2_ or the direct formation of carbamates. In a typical example, Lu et al. designed a sophisticated, one-step method for the synthesis of CO_2_-responsive iron oxide NPs in the presence of 1,8-diaminoocatne. About 8 nm of monodispersed iron oxide NPs were synthesized, and they exhibited low solubility in water (low zeta potential value). The system was purged with CO_2_, which resulted in the partial protonation of the terminal NH_2_ groups on the surface of NPs, which resulted in a large increase in zeta potential and a high colloidal stability in water, as shown in the Figure 6(ii) digital image. This was due to the terminal NH^3+^ groups with efficient hydration and a electrostatic double-layer repulsion-like charge on the NPs. Purging of N_2_ gas triggered the NPs re-assembly, and these eliminated the CO_2_ and increased the system pH to its initial value. DLS is used to monitor the reversible disassembly or assembly process; this results in an average NP size wave between 500 nm and 30 nm upon purging with N_2_ and CO_2_, respectively [84]. Variation in the hydrodynamic size of NPs in the presence of CO_2_ and N_2_ are shown in Figure 6(iii). The introduction of CO_2_ in the NPs not only increases the surface polarity but also triggers the assembly [85].

### 3.2. Physical Stimuli-Based Self-Assembly of Nanomaterials

Physical-responsive stimuli for the SA of NPs refers to the orientation of NPs by themselves under the applied physical means. This technique is also called an exogenous process, which means the force comes from the outside. Various types of physical stimuli include temperature, light, magnetic/electric field, and sound; these have been extensively used to control SA with a definite orientation. The SA of NPs via physical stimuli is one of the prominent techniques that can be applicable in vast range of applications ranging from biomedicine to electronic device fabrication.

#### 3.2.1. Light-Induced Self-Assembly

**Figure 7:**
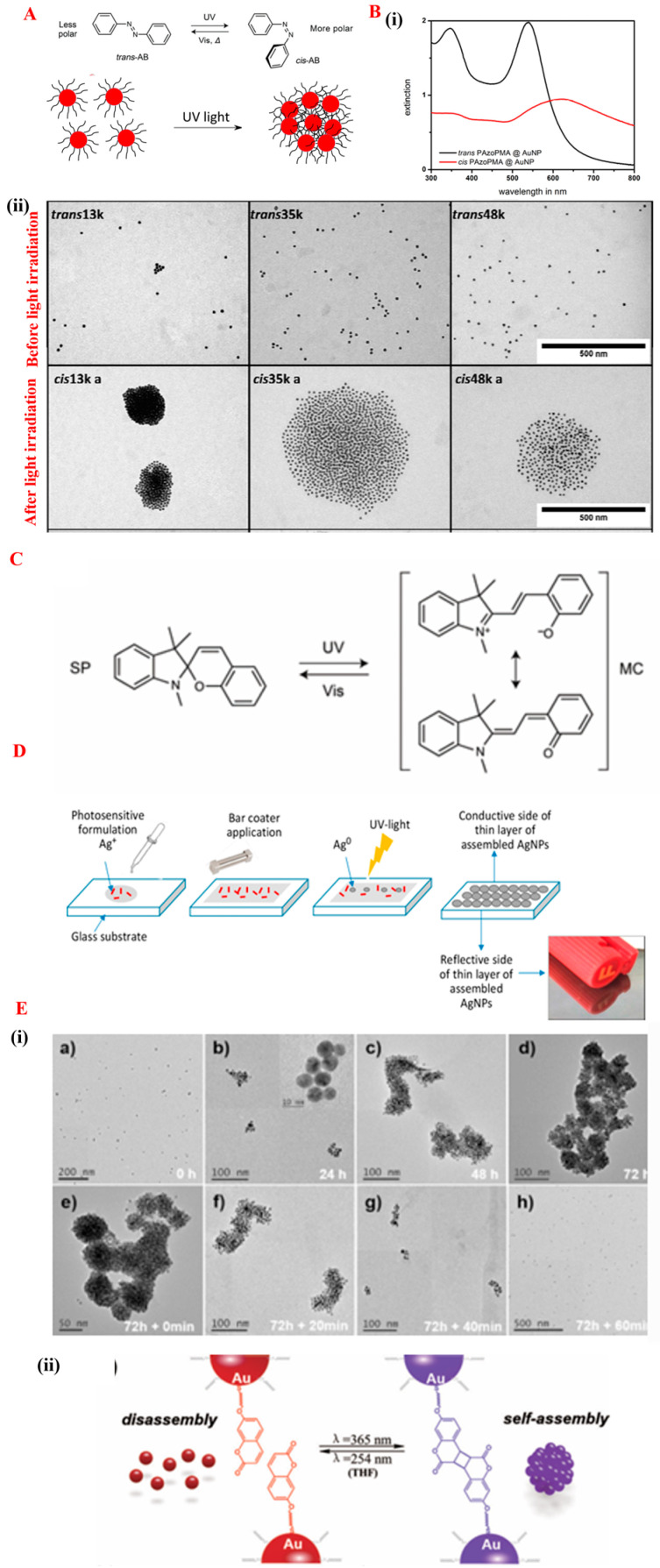
(**A**) represents the graphical representation of photo-responsive stimuli. The top image is cited and reproduced from the reference [91]. (**B**) (**i**) shows the change in the absorbance spectra of azobenzene-functionalized gold nanoparticles before and after photo-responsive stimuli (**ii**) HRTEM images of gold nanoparticles at different polymer chain lengths. This image was cited and reproduced from the reference [90]. (**C**) graphical representation of spiropyrne transformation to merocyanine and its reversibility under UV-Vis light. This image is cited and reproduced from the reference [91]. (**D**) development of mirror-like conductive surface on glass slide via light-induced assembly at liquid–air interface. This image was cited and reproduced from the reference [93]. (**E**) (i(a–d)) HRTEM images of coumarin-functionalized gold nanoparticle assembly and (**E**) (ii(e–h)) disassembly under light exposure. (**E**) (ii) represents the graphical representation of coumarin photo-response.

Light-responsive (photo-responsive) SA gained much attention among all the exogenous stimuli due to its economical, easy, and precise control over the intensity and remote delivery capability. Both monochromatic lights and polychromatic lights have been used as a light source to initiate the SA of NMs [86]. The SA of NPs can be achieved by using a photo-responsive surface functionality [87]. Traditionally, this process can be acquired by functionalizing the surface with a monolayer of photo-switchable molecules and the response can be tuned by the different wavelengths of light depending on the specific photo-sensitivity component on the surface of individual NPs. In general, light-responsive stimuli are a reversible process when the photosensitizers undergo photo-isomerization. The most commonly used photo-isomerization compounds include azobenzene, spiropyran, stilbene, diazonaphthoquinone, dithienylethene, and pheophorbide [88]. Amongst these options, azobenzene is a well-known and most-common photo-switchable component to acquire the SA of NMs upon irradiation with light. Azobenzene undergoes a transition that induces trans to cis transformation where nanomaterial agglomerate is controllable [89], and the respective graphical representation is shown in the Figure 7A.

In a classical example, the light-responsive methacrylic-type azobenzene monomer is grafted on the surface of gold NPs. UV-light irradiation at 365 nm for 30 min triggers the azobenzene moieties that induces trans-cis isomerization, which results in the aggregation of gold NPs in toluene. The transformation of trans to cis increases the polarity, and it leads to the formation of insoluble products. Additionally, it can also be useful for the switchable wettability that can be useful in various applications including the development of hydrophobic surfaces that help in self-cleaning technology. (Figure 6B left (i)) The red shift with an increase in the broadness of the plasmonic resonance peak of gold NPs confirms the photo-responsive SA of the nanoparticle. Notably, the interparticle spacing between self-assembled particles was influenced by the chain length of the grafted polymer. The before and after SA was shown in Figure 7B ((ii) top) and Figure 7B ((ii) down), respectively. For example, 13 k, 35 k, and 48 k molar mass polymer chains were grafted on the surface of gold NPs and irradiated under UV light, resulting in an increase in interparticle spacing [90]. Separately, spiropyran-functionalized nanoparticles are another example of a photo-induced SA technique. In this process, spiropyran undergoes light-induced isomerization via a spiropyran closed ring to open ring merocyanine isomer when irradiated under UV light and can be reversible in visible light (Figure 7C) [91]. Here, the spiropyran-functionalized NPs can be used in SERS (surface enhancement Raman spectroscopy) applications. The gaps raised due to the aggregation of nanoparticles during the SA acts a hotspot for SERS [92]. Recently, Etienne C. and co-workers developed a mirror-like conductive surface on a glass slide via a photosensitive formulation that contained a photosensitizer to generate free radicals upon exposure to UV light to induce SA. Authors have developed a self-assembled silver NP on a glass slide without using a stabilizing agent or polymer matrix. A photosensitive formulation that contained a mixture of silver nitrate and 2-hydroxy-2-methylpropiophenone was applied like a paste on a glass slide and irradiated under UV light to acquire the mirror-like conductive surface in a liquid–air interface, as shown in the Figure 7D [93]. This process can be advantageous to develop advanced light reflectors, solar cells, and smart windows.

Apart from the photo-isomerization and free radical process, the photo-cleavage technique is also one of the techniques used in SA processes. The word photo-cleavage refers the change in the structure of a molecule due to bond breakage when exposed to light. The most commonly used molecules are o-nitrobenzyl, pyrene, thymine, and coumarin; those undergo bond cleavage due to their interaction with UV light [88]. Amongst these options, coumarin was the most widely used photo-cleavage group. When compared to other photosensitive molecules, coumarin undergoes a cycloaddition reaction when exposed to a shorter wavelength. For example, coumarine-functionalized gold nanoparticles were self-assembled in a THF solution. When coumarine–gold NPs were irradiated under a 365 nm wavelength, the SA of gold nanoparticles in the THF medium was triggered (Figure 7E (top i(a–d)). Interestingly, the disassembly process was acquired by the exposure to even short wavelength light. The respective graphical images and high-resolution transmission electron microscopy (HRTEM) images were shown in the Figure (Figure 7E (down ii(e–h)) [94]. Thus, light-induced SA can be influenced by various parameters such as the solvent, ligand concentration, polymer chain length, light intensity, and irradiation time. The careful control of parameters during the manipulation of SA provides precise control over the size and density of NMs [86].

In the advancement of light-induced SA, optical tweezers are a versatile method to precisely control or manipulate the arrangement of NPs into ordered structures. Manipulation of micro- or nano-sized particles via optical forces gained notable attention among researchers in many fields of science. Generally, optical tweezers are a non-contact manipulation technique where a highly focused laser beam exerts a gradient force on an object, and it is capable of trapping and manipulating nano-sized particles [10]. In detail, when a laser beam focuses through the objective lens, the highest light intensity at a focal point experiences a gradient force that pulls particles towards itself. Once trapped, NPs moved and positioned in close proximity to other NPs through the optical tweezers technique facilitated electrostatic and van der Waals interactions between the NPs, resulting in the assembly of NPs into specific patterns. Therefore, SA patterns can be adjusted, or multiple traps can be acquired by moving the focal point to the specific site. On the other hand, the scattering force arises due to the reflection of light and pushes particles in the light propagation direction. Thus, the balance between scattering force and gradient force determines the stability of self-assembled nanostructures [95]. Generally, the manipulation and SA of metallic particles are highly challenging due to their high reflective nature that tends to push the particles far from the focal point. The choice of using an infrared laser that is far from the plasmonic resonance region of gold NPs made Svobada and Block trap 5 nm gold metallic NPs for the first time [96]. Non-spherical metallic NPs usually have a multiple plasmonic resonance that responds distinctly to polarized light. For instance, NRs show two plasmonic resonances that correspond to the oscillation of electrons in the longitudinal and transverse modes. Therefore, by controlling the polarization of the trapping laser, the orientation of NRs can direct the assembly of anisotropic NPs [97]. The precision and SA of metallic plasmonic NPs via the optical tweezers technique capitalizes on the significant advancements made in various applications; in particular, it creates hot-spots for light energy-harvesting applications, bioimaging, fabrication of data storage devices, and plasmonic meta-materials [10]. However, phototoxicity, surface adhesion, non-uniform force distribution, and the photothermal effect limits its application [98]. Optical tweezers generate significant heat when high-intensity laser lights were used during SA, resulting in damage or denaturation of sensitive biological samples or plasmonic materials [99]. A design for the fine-tuning and manipulation of the SA approach with reduced photo-thermal effect is beneficial when dealing with the SA of sensitive plasmonic metallic NPs and biological samples. Recently, C-shaped nano engraving has been used to reduce the photothermal effect and manipulate the NPs [100]. By controlling the polarization of the incident light, using C-structure engravings modulates plasmonic resonance and that helps to dynamically control the electric field, trapping more forces and plasmonic heating compared to the traditional method.

#### 3.2.2. Magnetic-Induced Self-Assembly

The precise control and guide of magnetic forces over the arrangement of tiny NPs into organized nanostructures under an applied magnetic field is known as magnetic field-induced SA. In this process, the core material of tiny particles that tend to assemble in the presence of a magnetic field should be ferromagnetic such as iron, nickel, and cobalt [101]. Magnetic forces provide an effective stimuli strategy that initiate easy and fast assembly, and they disassemble immediately when the field is dismissed [102,103]. This technique improves the magnetic nature of the material without physical manipulation or damaging the delicate samples. However, various parameters such as NPs size, NPs loading concentration, solvent, and the strength of the magnetic field influence these arrangements of nanostructures [104].

**Figure 8:**
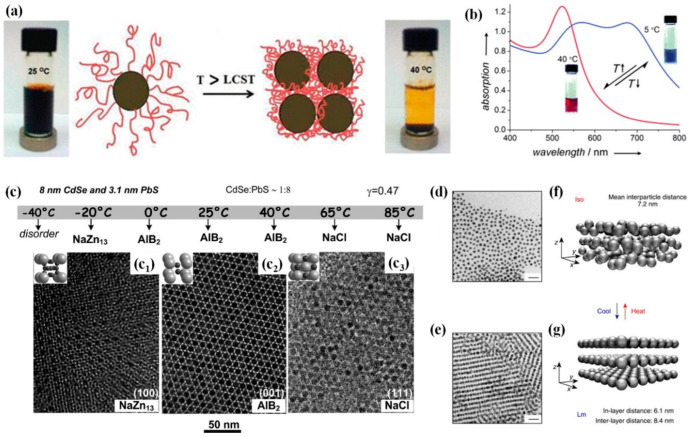
(**a**) lower critical solution temperature (LCST)-induced aggregation in water of individually coated magnetite poly(N-isopropylacrylamide) NPs. Adapted with permission from reference [119]. (**b**) The thermoresponsive tuning of plasmonic properties of charged colloidal AuNPs where the switching between the disassembled and assembled states in response to temperature change is observed. The changes in the LSPR are also shown. Adapted with permission from reference [120]. (**c**) Self-assembly of 8 nm CdSe and 3.1 nm PbS nanocrystals at different temperatures (c1) 40 °C (c2) 65 °C and (c3) 85 °C. Observation of the binary phases assembled at different temperatures. Typical electron microscopy images of binary superlattices isostructural with NaZn_13_, AlB_2_, and NaCl compounds are presented. Adapted with permission from reference [22]. Representative TEM image of thermally annealed Ag@L NPs presenting the patterns of (**d**) Iso and (**e**) Lm structures, scale bar, 20 nm. (**f**) Scheme of nanoparticle arrangements in Iso and (**g**) Lm structures as deduced from SAXRD and TEM. Adapted with permission from reference [121].

In a typical example, the SA of a cubic Fe_3_O_4_ particle was experimentally investigated at various parameters particularly by changing the orientation of the magnetic field and NPs at the loading concentration (Figure 8a). The author confirmed the formation of a 1D filament of short chains formed when 13.4 nm sized Fe_3_O_4_ cubes were placed under the magnetic field in non-miscible hexane solvents. Various superstructures such as helical, 1D filaments, and C-shaped SA have been developed by changing the concentration of Fe_3_O_4_ NPs [105]. In addition, relatively low magnetized materials such as cobalt or ferrite exhibit super ferromagnetic behaviors on self-assembled nanostructures. In a typical example, authors have prepared the FeCo/CoFe_2_O_4_ core shell structures and quantitatively evaluated the influence of the magnetic alignment and inter-intraparticle relation on improved collective magnetic characteristics. So far, most of the researchers focused on the magnetic field-induced SA of NPs using ferromagnetic/paramagnetic inorganic material or its composites [101]. However, developing a magnetic response in organic material is highly challenging due to their insensitivity to magnetic fields. In the year 2024, You-Jing. J and co-workers developed a magnetic response in organic materials by incorporating multiple magnetic field-sensitive materials in a single molecule. Utilizing the specific helix protein combined with the peptide bonds in a rod–coil block-like superstructure alters the magnetic response in organic materials [106]. However, in the utilization of only ferromagnetic/paramagnetic material, unwanted interactions limit this technique.

#### 3.2.3. Electric Field-Induced Self-Assembly

Electrical field-induced SA refers to the organization of NPs into ordered structures under the influence of an applied electric field. This SA technique on electric field-induced SA was less explored when compared with others because of their complexity of field design, scalability, and material constraints. The NPs should possess better dielectric properties to respond to the electric field. Here, the particles are trapped at the liquid–liquid interface under the influence of the electric field due to the capillary and electrostatic interactions [86]. Generally, uncharged particles agglomerate at the liquid interface to minimize its surface tension, but charged particles repel each other at the interface due to electrostatic interactions. Therefore, two immiscible liquid systems solve this problem where the liquid–liquid interface acts as an ion permeable under an applied electric field [87]. Dielectrophoresis, electrophoresis, and electrostatic interactions are the three main pathways to obtain the ordered self-assembled particles.

In the context of dielectrophoresis, a non-uniform electric field was used to control or manipulate the SA of particles into ordered structures. Depending on the variation in polarizability of particles and the surrounding medium, particles experience the positive or negative dielectrophoresis [107]. In detail, when a dielectric particle is placed under a non-uniform electric field, a dipole experiences unbalanced forces, causing particles to move either closer to or further away from the high electric field intensity. The SA of NPs in dielectrophoresis can be manipulated particularly by altering the electric field intensity, field distribution over NPs, size and shape of the particle, and polarizability of the medium [108]. The dielectrophoresis technique is not only useful for the non-contact manipulation and control of SA nanostructures, it can also be useful in the control and transport of micro-particles [109]. Non-contact separation makes dielectrophoresis advantageous in various fields such as microfluidics and biomedicine. In dielectrophoresis, the force extends for longer distances to trap the particle. However, due to a large area distribution of the electric field, the particle moves freely in the trap. Hence, the requirement of precise trapping for small-sized particles makes the dielectrophoresis technique less suitable for applications where accuracy is crucial. Therefore, developing and integrating a system which has both long-range forces to trap longer-distance particles and high-resolution systems that confine or hold the particle movement would be extremely valuable. In 2017, Mohammad Asif Zaman and co-workers demonstrated a method to trap NPs efficiently in small areas using a hybrid method. C-shaped engraving was used to create a small area to trap the particle and nanopillars were used to create dielectrophoresis forces, and it was confirmed that the probability trapping is better when compared to the conventional method. Apart from this, optoelectronic tweezers are one of the other proven versatile integrated tools in the manipulation and control of massive micro/nano SA structures that can be used in multiple applications [110,111]. These optoelectronic tweezers were developed to overcome challenges that limit conventional optical tweezers and dielectrophoresis. Optoelectronic tweezers utilize illuminated light to induce the electric field on photoconductive surfaces. Light-induced changes on the localized conductance exerts gradient electric field forces on particles in the medium [112]. Optoelectronic tweezers require low optical power when compared to conventional optical tweezers as light does not directly exert the gradient force for SA of NPs. A wide range of nanostructures can be created on the photo-conducting surfaces by altering the optical excitation profile. Most of the traditional optoelectronic tweezers utilize a uniform electric field and light to create a gradient force on photoconductive surfaces for the SA of NPs. These gradient forces on photoconductive surfaces allow for the positioning, trapping, and transport of particles for SA structures [113]. However, in a classical example, Mohammad Asif Zaman and co-workers demonstrated the SA of NPs through optoelectronic tweezers on a non-uniform electric field for effective trapping and SA nanostructures. A non-uniform electric field provides spatial variation in the electric field to enable the fine-tuning of particle movement [114]. This integrated optoelectronic system shows the capability to arrange NPs into linear chains and 2D lattice structures depending on the light pattern and configuration of the non-uniform electric field. On the other hand, combinatorial systems are widely used for the SA of complex structures. In a classical example, photovoltaic optoelectronic tweezers were used for the patterning of nanostructure ranges from clusters to grid-like structures. The patterning of NPs in photovoltaic optoelectronic tweezers was controlled by altering electric field parameters and illuminated light [115].

Electrophoresis is a technique where charged particles move towards and deposit on the oppositely charged electrode when the DC current is applied. The mobility and deposition of particles are mostly influenced by the size, molecular weight, and density of the particles [116]. Both of these techniques are mainly used in assembling biomolecules such as DNA, RNA, protein molecules, and cellulose. However, in electrostatic interactions, NPs that repel each other at the liquid–liquid interface due to their functionalized charged surfaces can assemble at two liquid interfaces under an applied potential. Montelongo and co-workers demonstrated this process experimentally by assembling the gold NPs at the water-1,2-dichloroethane interface by applying potential; 1,2-mercaptododecanoic acid stabilized gold NPs were dispersed in immiscible solvents. When a negative potential was applied (−0.2 V), the particle movement was noticed, and those gold NPs were self-assembled at the liquid–liquid interface. However, the reversibility of the self-assembled gold NPs was obtained by applying a positive potential of (+0.2 V) [117]. Thus, the electric field-induced SA of NPs can be controlled precisely, and it is reversible in nature.

#### 3.2.4. Temperature-Induced Self-Assembly

One of the important categories of SA via physical stimuli is the effect of temperature on the NP system [9,10,118]. In contrast to the electric field-, magnetic field-, and light-triggered SA of NPs, the temperature-driven approach comes with certain advantages. For instance, the NPs considered for SA using electric, magnetic, and light-based approaches should be intrinsically activated using certain ligands or molecules (biomolecules or macromolecules) that have an in-built response to the applied external field. Also, materials should necessarily be magnetically active for inducing the SA using the magnetic field. However, in the case of temperature-driven SA, the scope of utilizing the precursor is expanded to a large variety of NMs with a wide range of surface functionalities. This is because of a lesser dependance of the inherent physicochemical characteristics of the NPs on the applied temperature gradient. In this section, initially, we present a few examples of these temperature-driven SA experiments; following this, an interesting, recently developed technology based on cryosoret nano-engineering (CSNE) is presented in more detail [42].

Figure 8a presents a few representative examples of the SA of NPs achieved where the researchers considered modulating the temperature of the system under study. Balasubramaniam et al. demonstrated that the poly(N-isopropylacrylamide-*co*-Nile Red)-coated magnetic nanostructures assisted in the formation of SA upon subjecting the sample to the lower critical solution temperature (LCST) [119]. The obtained nano-assemblies demonstrated high water-dispersibility as well as enhanced transverse (T2) NMR relaxometric behavior as the temperature was increased through the LCST. This methodology of achieving the SA was utilized for monitoring the fluorescence intensity using the Nile Red co-monomer, which could enable the distinction of hydrated (nonaggregated) versus dehydrated (aggregated) states [119]. In another approach, Liu et al., presented an interesting approach to generate the thermoresponsive assembly and disassembly of charged AuNPs as shown in Figure 8b. Here, the changes in the temperature assisted in the realization of reversible assembly and disassembly of NPs, thereby yielding the dynamic and reversible modification of the surface plasmon coupling of the NPs [120]. In the typical experiment, the citrate-capped NPs covered with bis(p-sulfonatophenyl)-phenylphosphine (BSPP) were obtained using a ligand exchange process. Further, the appropriate addition of salt solution and agarose with associated heating conditions results in a final product that is thermoresponsive. Upon cooling to temperatures less than 40 °C, the viscosity increased with a concomitant formation of the hydrogel. The temperature can be modulated in order to obtain assembled (dark blue) and disassembled NPs (ruby red) [120].

In another study, Bodnarchuk et al. demonstrated an intriguing technique where the SA of monodispersed colloidal NCs has been achieved by varying the temperature as shown in Figure 8c. It has been shown that the formation of the superlattices is governed by the internal energy and entropy contributions to the overall free energy of the system under consideration [22]. The strong dependance of the formation of the binary superlattices on the temperature for mixtures of CdSe + PbS NCs and PbSe + Pd NCs has been demonstrated [22]. The emergence of high-density NaZn_13−_, AlB_2−_, and NaCl-type binary nanocrystal superlattices (BNSL) for CdSe and PbS NCs at different temperatures are captured in Figure 8c. From Figure 8c, we see that the mixtures of CdSe and PbS NCs were isostructural with NaCl-type binary superlattices at 85 °C; reducing the temperature to 65 °C resulted in the emergence of small domains of AlB2-type BNSLs. Further, subjecting the sample to solvent evaporation at 0, 25, or 40 °C resulted in the formation of AlB2-type BNSLs, reversibly [22]. Upon decreasing the temperature further, a NaZn13-type lattice was observed as shown in Figure 8c. As shown in Figure 8d–g, Lewandowski et al. demonstrated the SA approach using plasmonic AgNPs, where the NPs intrinsically had the thermally responsive organic coating to facilitate the SA [121]. The spatial distribution of the AgNPs can be reversibly switched on account of the dynamic SA resulting from temperature-driven changes of the organic coating shape. The TEM analysis of the samples presented interesting results where the Lm phase, the measured interlayer distance was 8.5 ± 0.4 nm (cooled), while for Iso the interparticle distance was 7.3 ± 0.8 nm (heated). A model was developed to understand the occurrence of the SA in the NPs, and it was noted that while the Iso phase presented a homogenous distribution of promesogenic and alkyl ligands, these entities were primarily located in between the layers for the Sm structure [121]. Such a spatial rearrangement of the NP metallic core assists in the formation of reversible SA structures. While these are the representative examples for the formation of SA by modulating the temperature, we present a close look into the recently developed technique based on the CSNE in the subsequent sections.

**Figure 9:**
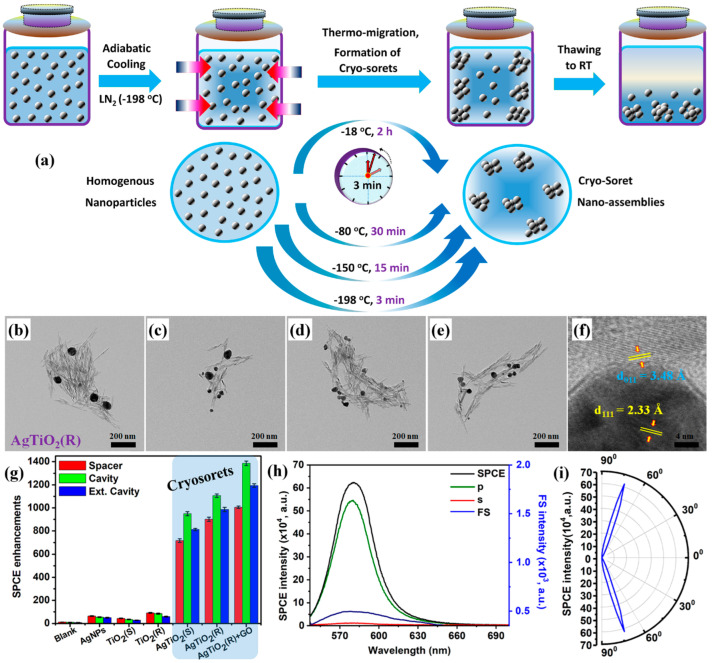
(**a**) The conceptual schematic of the cryosoret nanoengineering methodology. The reduction in the time required for the synthesis of the cryosorets with decrease in the cooling temperature is also captured. (**b**–**e**) Multiple TEM images of the AgTiO_2_ (rods, R) cryosorets (CS). (**f**) HRTEM image of the AgTiO_2_ (R) CS, where the d-spacing characteristic to that of AgNPs (shown in yellow) and TiO_2_ (R) (shown in blue) is presented. (**g**) SPCE enhancements realized with different types of nanomaterials in spacer, cavity, and ext. cavity nanointerfaces. (**h**) SPCE, FS, s, and p modes of the sample yielding highest SPCE enhancements shown with the corresponding angularity in Figure (**i**). Adapted with permission from reference [42].

Fundamentally, the CSNE is a technology where the homogenous solution of NPs is subjected to a temperature gradient to yield precise NAs. The temperature gradient assists in the migration of the NPs from the hot end of the system to the cold end through a phenomenon termed the soret effect [42,122]. Since the entire process can be carried out using cryo-environments (or low temperatures), the process of obtaining NAs through this technology is called CSNE and the thus-obtained assemblies are termed cryosorets. The schematic representation of the steps involved in the formation of the cryosorets is presented in Figure 9a, where the capability of low temperatures to yield cryosorets at faster rates is captured conceptually [42,122]. That is, while the process of obtaining cryosorets using −80 °C takes 30 min, the same process is completed in 3 min for a −196 °C temperature. In other words, at lower temperatures, the thermal gradient can overcome the electrostatic repulsion exhibited by the citrate-capped metallic NPs, resulting in precise nano-assemblies. It has been observed that while the freshly prepared (via the conventional Turkevich method using citrate as reducing and capping agents) plasmonic AgNPs are subjected to such low temperatures, precise NPs can be obtained, enabling researchers to tune the number of NPs per assembly by modulating the time of exposure to temperature gradient [42,122].

While the metal-dependent plasmonics have demonstrated several applications in the broad arena of photonics and biosensing, recently there is a growing interest towards engineering nanostructures with metal–dielectric hybrid interfaces to realize less lossy photo-plasmonic interfaces [123,124]. In this context, the cryosorets have been synthesized using high refractive index TiO_2_ NRs where the NRs are incorporated into the solution of AgNPs prior to exposure to the thermal gradient. The TEM images of obtained AgTiO_2_ cryosorets are shown in Figure 9b–e, with the HRTEM presented in Figure 9f, displaying the characteristic lattice fringes of Ag and TiO_2_.

The performance of the synthesized cryosorets was tested for enhancing the fluorescence signal intensity over the surface plasmon-coupled emission (SPCE) platform. The experiments were carried out in three configurations: (i) spacer, (ii) cavity, and (iii) ext. cavity nanointerfaces. In the spacer nanointerface, the cryosoret functions as a spacer between the metal thin film and the radiating dipoles. In the cavity nanointerface, the radiating dipole is sandwiched between the cryosoret and the thin metal film where the radiating dipole is in the cavity hotspot environment. The cavity is extended to a known distance in the ext. cavity nanointerface, where the cryosoret and the radiating dipoles are separated with a particular distance to avoid surface-induced quenching [38]. The experimental results of the SPCE enhancements obtained for studying these materials in spacer, cavity, and ext. cavity nanointerfaces are presented in Figure 9. The SPCE enhancements obtained with the use of different types of cryosorets are presented in Figure 9g. It is seen that the SPCE increased for all the samples as compared to the blank, indicating the effective nano-engineering of the SPCE interface assists in boosting the fluorescence signal intensity. While the plasmonic AgNPs yielded ~60-fold enhancements, the TiO_2_ spheres and rods presented ~50-fold and ~100-fold SPCE enhancements. Moreover, while such materials were utilized for the synthesis of cryosorets, >1000-fold enhancements in the fluorescence signal were observed. The SPCE, FS, p, and spectra along with the directionality data is presented in Figure 9h,i, respectively, for the sample yielding the highest SPCE enhancements in Figure 9g.

**Figure 10:**
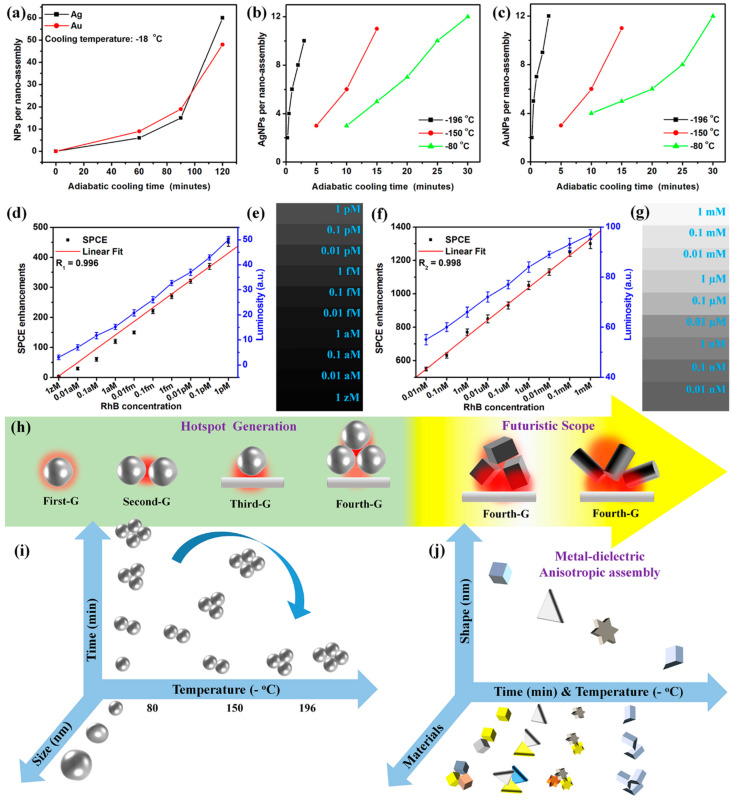
(**a**) AgNPs and AuNPs acquired per soret assembly with −18 °C adiabatic-cooling. (**b**) AgNPs obtained per soret assembly under −80, −150, and −196 °C adiabatic-cooling. (**c**) AuNPs obtained per soret assembly under −80, −150, and −196 °C adiabatic-cooling conditions. (**d**,**f**) Sensing of RhB demonstrating single-molecular limit of detection (zeptomolar). The luminosity values are plotted in the right *y*-axis along with the corresponding shade cards shown in (**e**) and (**g**), respectively. (**h**) Development of different generations of hotspots using CSNE technology. (**i**,**j**) Snapshots of the possible permutations and combinations of cryosoret-based nanoengineering that the current research work presents to the wide community of nanoscience and photoplasmonics. Adapted with permission from reference [4].

Figure 10 presents an executive summary of the key research highlights of CSNE technology. From Figure 10a–c we see that the number of NPs forming the assembly increases with the decrease in the temperature (−18 °C to −80 °C to −150 °C to −196 °C) for plasmonic AgNPs and AuNPs. This indicates that NP assemblies can be obtained at reduced time intervals by using lower temperatures. Further performance of the synthesized AgTiO_2_ (R) CS was tested for sensing RhB at trace concentrations, where RhB serves as an SPCE reporter molecule. From Figure 10d–g we observe that a linear relationship is observed for the RhB sample sensing with zeptomolar limit of detection. This high sensitivity is attributed to the metal–dielectric hybrid coupling of the AgTiO_2_ (R) CS with the surface plasmon polaritons (SPPs) of the metallic thin film. Further, the obtained emission was captured using a smartphone camera and analyzed using a simple Color Grab app to obtain the luminosity values. It is also seen that there is an excellent correlation between the SPCE enhancements and the luminosity values, thereby emphasizing that the sensing can be effectively performed using a cost-effective smartphone-based detector platform.

Furthermore, it is important to comment upon the effectiveness of the fourth generation of plasmonic hotspots that are successfully demonstrated using the CSNE protocol (Figure 10h). The plasmonic NP with an ability to confine the incident EM radiation to local regimes on account of the LSPR effect comprises the first generation of plasmonic hotspots. Further, the EM field intensity significantly increases in the nano-junctions between the plasmonic NPs, in typical configurations such as nano-dimers (metal–metal, metal–dielectric, dielectric–dielectric), and this comprises the second generation of hotspots. The plasmonic coupling between the localized surface plasmon resonance (LSPR) of the NP and the SPPs of thin metallic film results in the generation of the third generation of plasmonic hotspots in a typical nanoparticle-on-mirror (NPoM) configuration. In this background, the fourth generation of plasmonic hotspots comprises the hybrid coupling of modes sustained by the nano-assemblies with that of the modes sustained by the metallic thin film, when appropriate phase matching conditions are satisfied. The CSNE demonstrates an effective methodology to integrate the main three sub-domains of plasmonics: (i) metal plasmonics, (ii) dielectric plasmonics, and (iii) graphene plasmonics, as elaborately presented in the related publication [33,123,125]. Figure 10i,j presents the overview of the futuristic scope and opportunities rendered by the CSNE technology where altering the shape, size, and material properties of the starting materials would render desirable properties for applications concerning plasmonics, optoelectronics, biosensing, and molecular diagnostics.

## 4. Futuristic Scope and Perspectives

From the dynamic SA found in planetary motion to the self-regulated SA in biomolecular machines in living organisms, the process conditions and fundamental principles of SA have always motivated researchers in the broad domains of nanoscience and nanotechnology [9,10]. While the futuristic scope and perspectives are broadly dependent on the current technological evolutions in the domain of SA, the crucial aspect of drawing inspiration from the elements of nature cannot be ignored. For instance, the non-pigmentary coloration of the scales in butterflies, fishes, and birds have triggered the transformation of optical chips and related platforms’ photonic crystal frameworks with augmented performance [5,126,127]. Similarly, there are several examples in which researchers and scientists draw inspiration from observations made from the living and non-living world around us. While this remains a wider territory for future research, it is instructive to briefly present key highlights in this direction [24,79,118].

While there are different methodologies adopted for the realization of SA in nanostructures, the permutations and combinations of the existing methodologies would present new rooms for achieving desired functionalities. Such nano-engineering would need meticulous design and synthesis of SA of NPs, where the fundamental principles of organic chemistry, materials science, optoelectronics, and biocompatibility need to be analyzed. Although the existing NA synthesis technologies aid the researchers in exercising the liberty to fabricate desirable NAs of hybrids of materials from the s, p, d, and f block elements of the periodic table, certain aspects in nano-engineering need to be accentuated. At this juncture, we would like to highlight the aspect of channeling the focus of nanoscience research, especially in the domain of NA fabrication, towards recognizing and incorporating the core principles of green nanotechnology [128,129,130]. This aspect is of paramount significance on account of the perilous effects the synthesized NMs have caused in the past few decades. Even as the field began to mature with several publications and patents in the broad domain of nanoscience and NA, little attention has been paid towards the toxicological effects of the synthesized materials [131]. While fundamental NA approaches can be categorized into bottom-up and top-bottom routes, the possibilities and the experimental feasibility to combine materials at nanoscale using different NA routes are infinite, which demands the researchers to re-evaluate the pertinence of the approach from the perspective of green nanotechnology principles.

The generation of SA of NMs with unique functionalities can assist in the realization of new states of coupling such as exciton–exciton coupling, plasmon–exciton coupling, plasmon-fluorescence resonance energy transfer (FRET) coupling, and magnetic plasmon-electric plasmon coupling [132,133,134,135,136]. In this regard, several research groups across the globe are searching for materials with unique optoelectronic and magnetic properties, which has resulted in numerous publications. Recently, the focus is being shifted towards departing from lengthy and laborious lab-confined experiments to software-based simulation tools to understand the systems’ performance at least semi-quantitatively. In this regard, numerous iterations with respect to material properties, size, shape, and the nearby environment have been explored to obtain a prerequisite understanding prior to performing the associated experiments in the wet-lab. Although such simulations and theoretical models provide an adequate understanding of the systems under consideration, the expected outcomes in the software-based models can be significantly different from the ones observed experimentally on account of the approximations made in the former scenario, often to a large margin. In this regard, there is a growing demand to utilize the understanding from the large data sets where the key highlights and learning from reported works can be fed to a self-learning tool to comprehend, analyze, and evaluate the best possible configuration for experimental work. With the advent of advanced artificial intelligence tools, appropriate materials with desired properties can be categorized for the synthesis of SA structures. For instance, AI and machine learning-based algorithms can be designed to evaluate the performance of the different types of coupling phenomena between different types of materials at nanoscale dimensions using relevant tools in the computer system before carrying out experiments in lab-based setups. Nanotechnology and related explorations are already being integrated with AI and machine learning tools in myriad fields of applied sciences such as agriculture [137], scalability [138], plasmonics [139], nanomanufacturing [140], diagnostics [141], materials discovery [142], and precision cancer medicine [143]. In this background, we would like to emphasize that there are several future opportunities in the broad domain of SA and related technologies, especially at the intersection of AI-based tools to streamline the SA approaches and lab-driven experimental technologies.

Further, although there are numerous reports highlighting the utility of NAs for diverse applications, it is important to note that the mechanism of formation of different NAs with the use of different sizes, shapes, heterogeneity, and polycrystalline natures is yet to be explored. Moreover, it is expedient to shift the research direction from a more general approach where the obtained NAs are classified as dynamic or static, to a more focused approach where the actual role of thermodynamic and kinetic processes needs to be elaborately discussed. Additionally, there are several examples of SA processes that cannot be categorized as either stimuli-dependent or stimuli-independent. This vague type of SA often depends on the combination of external stimuli and intrinsic properties of a material. For instance, a thermo-responsive polymer poly(N-isopropylacrylamide) generally self-assembles in an aqueous medium. However, its self-assembly and disassembly depends on the temperature. Though it exhibits a thermal response SA at a low critical solution temperature, some of the thermo-responsive polymers cannot be categorized into stimuli-responsive or stimuli-dependent techniques due to their intrinsic behaviors [144]. Similar to thermo-responsive materials, some of the magnetic field-responsive NA also cannot be categorized into stimuli-responsive or stimuli-independent SA. For example, super-paramagnetic iron oxide NPs can SA directly due to dipole–dipole interaction and external stimuli directs super-paramagnetic NPs effectively to form complex SA nanostructures [145]. Therefore, NA in super-paramagnetic NPs cannot be categorized. Block copolymers like poly(acrylic acid)-block-polystyrene SA organize into vesicle or micelle nano-structures depending on the amphiphilic behavior of the polymer. However, reversibility can be directed by changing the ionization through varying the pH in the surrounding medium, as block co-polymers SA without the interference of external stimuli and re-assemble or disassemble under the influence of pH, solvents, or temperature [146]. This vague type of SA cannot be classified as either stimuli-dependent or stimuli-independent SA techniques. Although these vague-type SA techniques are advantageous smart-responsive technologies, their complexity, inconsistent outcomes, and unpredictability limit their application where precision plays a crucial role. Hence, strong attention has to be focused on controllable and established self-assembly mechanisms. Moreover, the appropriate characterization with adequate use of in situ and operando technologies need to be employed and developed to comprehend the exact nucleation and formation of NAs. More emphasis needs to be placed on the realization of simple, direct, and highly reproducible synthesis approaches for NA generation via a controlled fashion, especially in the cases concerning highly organized, complex, and self-assembled hierarchical structures. Such extensive analysis of the pre-programmed and robust NAs would render the developed technology promising for exploration in spintronic devices, metamaterials, photovoltaics, sensing, photo-plasmonics, and imaging applications.

Further, it is important to discuss the limitations of this review. In this review we attempt to provide a comprehensive overview of self-assembly, yet it is important to acknowledge that the vastness of this field precludes an exhaustive treatment of all relevant examples. Specifically, the self-assembly of low-dimensional substrates such as 0D (carbon dots), 1D (CNT), and 2D materials (such as graphene and their analogous for instance), with a particular focus on carbon-based materials, represents a crucial area of interest within nanoscience and nanotechnology with multifaceted applications. An example of this is the way in which the self-organization of carbon-based nanomaterials, such as graphene, carbon nanotubes, and other allotropes, advances our understanding of material science and technology from an applied science standpoint. Carbon nanodots and their hybrids with other materials have been used to develop mechanically robust macroscopic functional substrates [147] useful in theragnostic [148], imaging, and sensing applications [149,150]. Next-generation high-performance transistors in the semiconductor roadmap have been achieved using the self-assembly of 1D carbon nanotubes [151,152], and the self-organization of other carbon-based materials has been utilized for applications including but not limited to optoelectronics, photocatalysis, energy generation, and water treatment, to name a few [153,154,155,156,157,158]. While these topics are pivotal from both fundamental and applied science perspectives, they are not covered in extensive detail within this review. In this regard, we would like to direct readers to associated literature that delves deeper into these areas.

## 5. Conclusions

SA processes are ubiquitously observed in the nature around us in living and nonliving systems. By and large, the SA of NMs is driven by the attainment of a globally ordered system from a less-ordered state, via certain principles that are locally operational and regulated in the micro–macro systems. The tendency to attain the energetic minimum state, which is also the most stable existence of any system, is facilitated by several aspects of SA, including the nano-components of interest, the interactions at the surface level between the considered NPs, the reversibility of the processes involved, and the nature and chemical functionality of the immediate nano-environment, as well as the flexibility to re-orient via controllable mobility. This review presents a comprehensive understanding of the different types of SA techniques that are available, where we initially introduce the classification and then elaborately discuss a few case studies.

In summary, this review focuses on two major approaches of obtaining SA of NMs, including the stimuli-independent and dependent methods. While we detailed the solvent evaporation and gravitational sedimentation methods as stimuli-independent approaches, the stimuli-dependent ones were further classified as physical and chemical stimuli-based techniques. The light, electric-field, magnetic-field, and temperature-driven SA of NMs were discussed under the umbrella of physical stimuli-based approaches. The solvent, acid–base, metal ion, biomacromolecule, and gas-induced SA of NMs were captured and discussed in the domain of chemical stimuli-based SA. While we present a broad overview of the different techniques and mechanistic understanding of each of these categories, it is important to note that the successful incorporation of SA approaches in semiconductor devices as well as microelectromechanical (MEMS) technologies is expected to become a reality with the advent of novel nano-engineering protocols that are being developed and refined incessantly. The research highlights captured in this review are expected to serve as a guiding tool for researchers in the field of nanotechnology, especially catering to longsighted applications related to lab-on-a-chip, lab-on-molecule, and related applications associated with the internet-of-things (IoT). The comprehensive coverage of the key highlights from the existing techniques for SA would foster further fundamental research in this direction, suitable for applications in the intersecting fields of physics, chemistry, and life sciences.

## Abbreviations

APTES	3-Aminopropyltriethoxysilane
AI	Artificial Intelligence
BNSL	Binary Nanocrystals Super Lattices
BSPP	Bis(p-Sulfonatophenyl)-Phenyl Phosphine
CTAB	Cetyl Trimethyl Ammonium Bromide
CSNE	Cryosoret Nano-Engineering
DMSO	Dimethyl Sulfoxide
DCM	Dichloromethane
DDA	Dodecylamine
DNA	Deoxyribonucleic Acid
EDTA	Ethylenediaminetetraacetic Acid
FRET	Fluorescence Resonance Energy Transfer
HAADF-STEM	High-Angle Annular Dark-Field Scanning Transmission Electron Microscopy
HSNs	Hollow Spherical Nanostructures
HPA	Hexylphosphonic Acid
HRTEM	High Resolution Transmission Electron Microscopy
IoT	Internet-of-Things
LSPR	Localized Surface Plasmon Resonance
MEMS	Microelectromechanical
NPOM	Nanoparticle-on-Mirror
NPs	Nanoparticles
NMs	Nanomaterials
NCs	Nanocrystals
NA	Nano-Assembly
NRs	Nanorods
OA	Oriented-Assembly
Olam	Oleyamine
ODPA	Octadecyl Phosphonic Acid
PS	Polystyrene
P4VP	Poly(4-Vinyl Pyridine)
PBuA	Polybutyl Acrylate
RhB	Rhodamine B
SA	Self-Assembly
SPCE	Surface Plasmon-Coupled Emission
SPPs	Surface plasmon polaritons
SAED	Selected Area Electron Diffraction
SAM	Self-assembly monolayer
SERS	Surface-Enhanced Raman Scattering
TPs	Tetrapods
TEM	Transmission Electron Microscopy
THF	Tetrahydrofuran
T-DDT	t-dodecanethiol
1D	One Dimensional
2D	Two Dimensional
3D	Three Dimensional
